# A Unique Case of Pneumatised Styloid Process with Cholesteatoma

**DOI:** 10.1155/2013/964725

**Published:** 2013-06-04

**Authors:** Mostayn Alam, Raghav C. Dwivedi, Faruque Riffat, Daniel Scoffings, David Moffat, Piyush Jani

**Affiliations:** ^1^Department of Otolaryngology, Head and Neck Surgery, Addenbrookes Hospital, Cambridge University Teaching Hospitals NHS Foundation Trust, Cambridge CB2 0QQ, UK; ^2^Department of Radiology, Addenbrookes Hospital, Cambridge CB2 0QQ, UK

## Abstract

Pneumatisation of styloid process is a very rare finding and has never been reported previously. We present a unique case of a pneumatised styloid process with a cholesteatoma arising within the cavity. We describe the clinical features, associated radiological findings, and management of this lesion.

## 1. Introduction

Styloid process develops from the dorsal part of the second branchial arch (Reichert cartilage) at around fourth week of intrauterine life [1, 2]. Its base becomes embedded in the petromastoid region of the temporal bone thereby making it a part of temporal bone. The pneumatisation of temporal bones starts late in fetal life and continues until the adult life [3]. Pneumatisation process is variable and in cases where there are well pneumatised temporal bones, accessory air cells may occur and communicate with the middle ear mastoid air cells [3, 4]. Therefore theoretically pneumatisation can be seen in some portions of the skull base as well [3]. Extensive pneumatisation of skull base and atlas vertebrae [3] has been described in the literature; however, pneumatisation of the styloid process has not been reported before. We present a unique case of pneumatised styloid process with a cholesteatoma arising within it. 

## 2. Case

A 43-year-old female presented to our otolaryngology department with an 11-month history of recurrent, right-sided otalgia and foul-smelling otorrhoea. The condition had been misdiagnosed as recurrent otitis externa. Patient denied any history of hearing impairment, vertigo, tinnitus, facial nerve palsy, or a history of head trauma. Clinical examination revealed squamous debris and purulent material in a grossly eroded floor of bony meatus with the lower half of the tympanic membrane hanging in the breeze. The facial nerve function was normal and there were no other neuro-otologic signs. Examination of contralateral ear and rest of the ENT examination were unremarkable.

**Figure 1:**
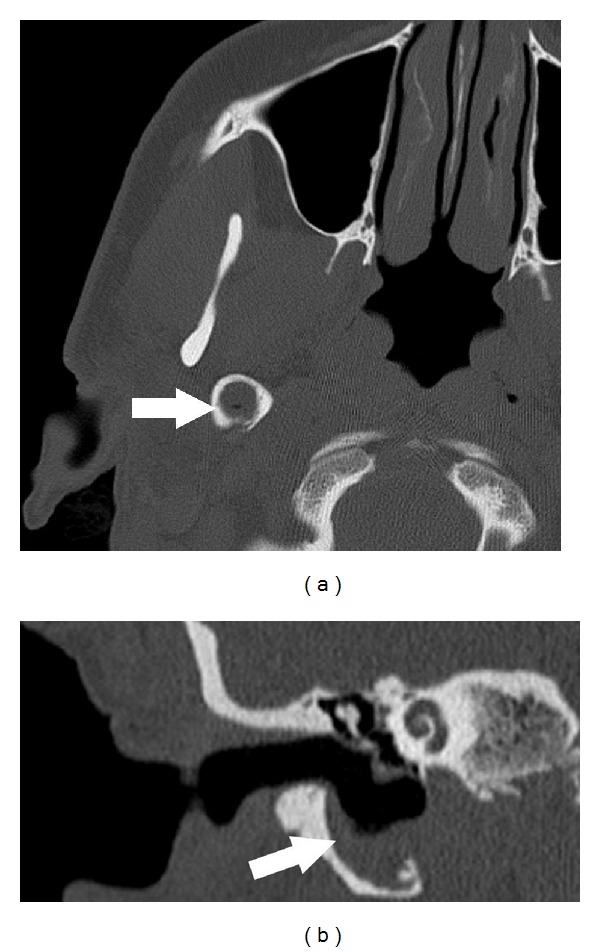
(a) Axial CT shows soft tissue attenuation material filling an expanded and pneumatised right styloid process (arrow), which on coronal reformat (b) communicates with an expanded hypotympanum.

**Figure 2:**
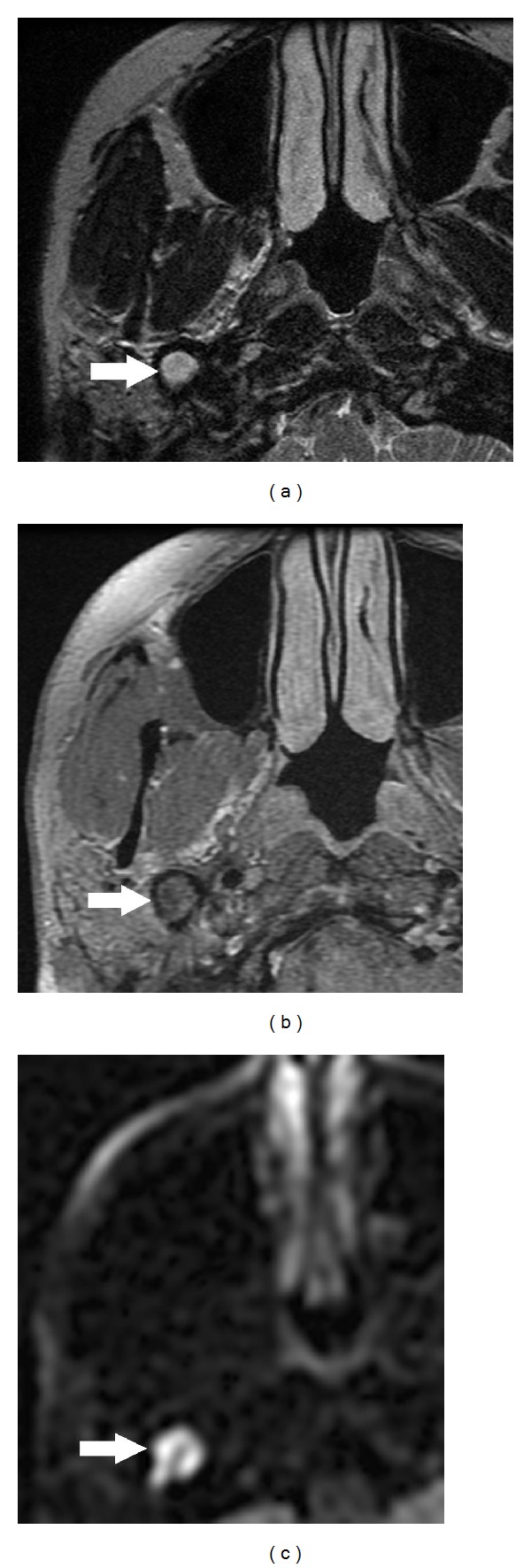
(a) MRI shows that the material in the pneumatised right styloid process is hyperintense on T2-weighted imaging (a), of intermediate signal intensity on fat-suppressed T1-weighted imaging (b), and is markedly hyperintense on PROPELLER (periodically rotated overlapping parallel lines with enhanced reconstruction) diffusion-weighted imaging (c). These signal characteristics are typical of cholesteatoma.

CT scan of the temporal bone demonstrated an extensively pneumatised styloid process containing a soft tissue mass (Figures 1(a) and 1(b)). Further MRI imaging showed a hyperintense material in the pneumatised right styloid process on T2 weighted imaging (Figure 2(a)), of intermediate signal intensity on fat suppressed T1 weighted imaging (Figure 2(b)) and marked hyperintensity on PROPELLER (periodically rotated overlapping parallel lines with enhanced reconstruction) diffusion weighted imaging (Figure 2(c)). These signal characteristics are typical of cholesteatomas. In addition there was erosion of the hypotympanum. No intracranial involvement was seen. Pure tone audiogram revealed mild conductive hearing loss in the right ear with threshold of 30 db and normal thresholds on the left side. 

The patient subsequently underwent a right combined approach tympanoplasty with extended posterior tympanotomy approach continuing the dissection inferiorly to the hypotympanum and down to the styloid apparatus. The lesion in the pneumatised styloid process contained a fibrous cyst wall full of cholesteatoma. This was completely excised and the cavity was obliterated with fat. Cartilage was used to reconstruct the defect in the floor of the bony external auditory canal. There were no postoperative complications and recovery was uneventful. Patient remains disease free until the last followup (14 months after surgery). 

## 3. Discussion

The exact gestational stage at which pneumatisation of temporal bone starts is controversial; however, it is thought to start sometime between 24 and 38 weeks in utero and then continue into adult life [3, 4]. Pneumatisation of temporal bone can be divided into three stages: infantile, transitional, and adult corresponding to the ages 0 to 2 years, 2–5 years, and into adult life, respectively [3, 4]. The degree and areas of pneumatisation of temporal bone vary enormously from person to person and have been reported in 14%–96% of all adults [4]. The pneumatised air cells are lined by single layer of squamous epithelium which is the continuation of the middle ear lining [3, 4].

Anatomically the pneumatisation of temporal bone can be divided into five regions, mastoid, middle ear, perilabyrinthine, petrous apex, and accessory region. The detailed description of temporal bone pneumatisation is beyond the scope of this paper and is well described by Virapongse et al., 1985 [4]. It is theoretically possible for the accessory air cells in the skull base to extent into the styloid process [4]; however, this has never been reported previously. 

Pneumatisation of temporal bones is thought to be necessary for lightening the weight of the skull, providing a reserve volume of air (air reservoir action), insulation, reception, resonance, and acoustic dissipation of sound. Any alteration of the reservoir function of temporal bone and eustachian tube dysfunction leads to generation of negative pressure within the middle ear culminating into retraction of tympanic membrane and cholesteatoma formation [3, 4]. 

Cholesteatoma most frequently involves the middle ear and mastoid but may develop anywhere within the pneumatised portions of the temporal bone including the petrous apex [5]. The literature reports cholesteatoma at some unusual sites such as frontal [6], maxillary, and ethmoid sinuses [7]. Cholesteatoma within pneumatised styloid process presented here is yet another addition to the list of unusual sites.

By the nature of the disease cholesteatoma causes destruction of the surrounding bone and local erosions which at times may be extensive [8]. It is not uncommon for a middle ear cholesteatoma to cause erosion of the hypotympanum [9]. However, occurrence of cholesteatoma in the pneumatised styloid process and extending into the hypotympanum is very unusual and has not been reported in the literature. We believe that in our patient the pneumatisation of the styloid process aided in the development of the cholesteatoma and its extension into the hypotympanum (Figures 1(a) and 1(b)).

Diagnosis of cholesteatoma is made clinically although radiology is helpful in difficult cases as described here. CT and MRI with their various sequences (T1 weighted, fat suppressed T2 weighted, and PROPELLER diffusion weighted imaging) complement the clinical examination [8] as in our case and confirm the extent of cholesteatoma (Figures 2(a), 2(b), and 2(c)). They also help with operative planning.

Surgical resection is the management of choice for both acquired and congenital cholesteatoma. The two primary goals of surgery are to remove the disease whilst managing complications and to reconstruct the middle ear. Prognosis of both congenital and acquired cholesteatoma is generally good.

## 4. Conclusion

In summary, we present the first reported case of pneumatisation within the styloid process. Unusually, the patient in this case also developed cholesteatoma within the cavity. Pre-treatment scanning of the temporal bone and stylomastoid apparatus should always be performed before planning the surgery so as to minimize chances of leaving behind the residual disease at unusual places of pneumatisation of temporal bone including the skull base.
